# Multi-Biometric System Based on Cutting-Edge Equipment for Experimental Contactless Verification

**DOI:** 10.3390/s19173709

**Published:** 2019-08-26

**Authors:** Lukas Kolda, Ondrej Krejcar, Ali Selamat, Kamil Kuca, Oluwaseun Fadeyi

**Affiliations:** 1 Faculty of Informatics and Management, University of Hradec Kralove, Hradec Kralove 50003, Czech Republic; 2 Malaysia Japan International Institute of Technology (MJIIT), Universiti Teknologi Malaysia Kuala Lumpur, Jalan Sultan Yahya Petra, Kuala Lumpur 54100, Malaysia; 3 Media and Games Center of Excellence (MagicX), Universiti Teknologi Malaysia, Skudai 81310, Malaysia; 4 School of Computing, Faculty of Engineering, Universiti Teknologi Malaysia (UTM), Skudai 81310, Malaysia; 5 Department of Geology, Faculty of Space and Environmental Science, University of Trier, 54296 Trier, Germany

**Keywords:** biometry, identification, bloodstream, image recognition, multi-biometrics

## Abstract

Biometric verification methods have gained significant popularity in recent times, which has brought about their extensive usage. In light of theoretical evidence surrounding the development of biometric verification, we proposed an experimental multi-biometric system for laboratory testing. First, the proposed system was designed such that it was able to identify and verify a user through the hand contour, and blood flow (blood stream) at the upper part of the hand. Next, we detailed the hard and software solutions for the system. A total of 40 subjects agreed to be a part of data generation team, which produced 280 hand images. The core of this paper lies in evaluating individual metrics, which are functions of frequency comparison of the double type faults with the EER (Equal Error Rate) values. The lowest value was measured for the case of the modified Hausdorff distance metric - Maximally Helicity Violating (MHV). Furthermore, for the verified biometric characteristics (Hamming distance and MHV), appropriate and suitable metrics have been proposed and experimented to optimize system precision. Thus, the EER value for the designed multi-biometric system in the context of this work was found to be 5%, which proves that metrics consolidation increases the precision of the multi-biometric system. Algorithms used for the proposed multi-biometric device shows that the individual metrics exhibit significant accuracy but perform better on consolidation, with a few shortcomings.

## 1. Introduction

Due to the rapid development in information technology, it has become possible to utilize biometrics for identifying and verifying persons [1,2,3]. Personal verification involves associating an identity with a specific individual. Verification or authentication of an identity is related to the authorization or refusal of an individual’s personal identification, which is verified and confirmed side-by-side with the identity provided. This procedure is crucial for identifying a search query of a person.

In recent times, biometric verification and identification systems have gained popularity, which brought about their extensive usage [4]. Most significantly, it is common to see laptops with fingerprint readers, as well as a Windows 10 “hello” function, which both support biometric identification and verification [3,5,6,7]. The latter feature is available to users who sign up for biometry usage. Biometry saves the user the stress of regular logins into gadgets and wares (e.g., keys or cards) with privacy and identity theft challenges [8]. For instance, lost or forgotten login details can be accessed by a third party and be used illegally. Biometry eliminates such problems, and greatly reduces the risk of copying or falsifying them. Nevertheless, biometry is not a complete and perfect solution for user verification and identification. Gathering of an individual’s biometric data to create the person’s biometric template is a complex process, which can sometimes yield an indefinite outcome [9]. However, the probability of success with biometric systems may vary up to 99% in the best systems. As such, biometric systems are greatly beneficial in curbing security challenges such as privacy invasion and identity theft problems (the most common being attacks carried out using dummies or models of the given body part).

Hand recognition remains the earliest form of available biometric characteristics used to identify and differentiate humans, as well as verify their identities [10]. Using the hand, it is possible to recognize, for example, hand geometry, fingerprints, palm lines, lines on finger joints, and image of the bloodstream. [11]. As soon as a person attains adulthood, features of the individual’s hands remain the same for the rest of his or her life. Hence, these characteristics can be used to identify and/or verify the person [12]. Moreover, these characteristics can be scanned (excluding the fingerprint) using a camera with a basic resolution (640 x 480 pixels), and saved in biometric memory, unlike the scanning of the eye retina [13] (which may not necessarily be saved) for future references. From the foregone, we can say that it is safe and inexpensive to embark on a project of creating small devices for the scanning biometric characteristics of the hand.

A number of biometric systems currently exist that work based on the principle of the shape and surface elevation of the hand (jointly referred to as “hand contour” in the context of this work), and serves the purpose of a person’s identity features [14,15,16,17,18,19,20]. The design and implementation of such systems offer several benefits, especially given that hardware and software requirements are very easy to come by. However, biometric characteristics of a hand contour is often not sufficient to distinguish individuals. Consequently, identification security is compromised, which leads to a high false-match rate (FMR). As a result, optimized biometric systems are continuously designed based on the biometric characteristics that are hidden, and cannot be replicated. For example, ultramodern finger print scanning results cannot be replicated, but the cost associated with its design and implementation is very high. A second possibility for improving identification and verification by biometric systems is the use of two or more biometric characteristics within a system. For instance, joint scanning of hand and blood stream offer better identification results, which, in recent times, have become a lot cheaper, and have improved their security [21]. This is enabled by the fact that false match rates (FMR) of the individual biometric characteristics are multiplied, which, in turn, decreases FMR. Hence, the whole system becomes more secure.

### Related Literature

As development in science and technology progresses, smarter ways of operating household and office equipment are becoming more popular. For instance, Reference [20] described a gesture-based technology that makes use of some specific grammatical terms in its operation. This is possible due to these technologies and continuous issues of fake identity behind finger print detection [22] and walking patterns (also known as “gait”) [23].

According to Reference [24], traditional singular biometry identifiers are flawed due to varying interclass sensitivity, data that bears a lot of noise, or very high error margins. For instance, in today’s world of mobile phones, Reference [25] tried to analyze using a mobile phone, hand segmentation, and fingertip readings of about a hundred subjects. It was observed that the sensitivity of the technique yielded around 52%, a figure which may have been more precise if the method was bi-biometric-based or multi-biometric based. As a result of the sensitivity shortcoming common to uni-biometric systems, Reference [24] proposed a multi-model technique by adopting fused faces and fingerprints, i.e., making use of a collaboration-based classifier to identify the faces of a different person. A selective neural network was jointly used with “Viola–Jones method-based PCA” by Reference [26] for a gridding system. Here, over 100 faces from a face-based database were distinguishable, which proves the success of the method. In comparison to the work of References [25,27], the merged vascular recognition with hand geometry in a multi-modal design derives an equal error rate of 6%. While singular biometric techniques are currently less preferred, the unique and greater sensitivity of multi-biometric technologies seem to be revolutionizing the field of biometry. In a fascinating technological twist, biometry has stepped forward beyond mere gesture identification to commence blood-based verification using physiological features, as reported by Reference [28]. Table 1 summarizes a few multi-modal biometric studies (within the last 10 years) with their corresponding error rates. From the table, HG denotes hand geometry, FV denotes finger vein (vascular structure), PP is the palm print, MFV is the multi-finger vein, FK is the finger knuckle, KS is the knuckle shape, and FG is the finger geometry.

Except otherwise stated, the following performance ratings (abbreviations) have been used throughout this paper and in similar biometric literature.

▪ EER (Equal Error Rate)
▪ FAR (False Acceptance Rate)
▪ FRR (False Rejection Rate)
▪ FIR (Far infrared)
▪ NIR (Near Infrared)
▪ ROI (Point or region of interest on the image)

The rest of this paper is organized as follows. Section 2 further describes the problems upon which the study lies, and for which more multi-modal techniques are required. Section 3 describes in detail the newly proposed multi-biometric method, while experimental testing of the system and discussion of the generated results are reflected in Section 4. The final section discusses the limitations of the new method and suggests ways to improve the limitation in future research studies.

## 2. Problem Definition

Users’ hand-geometry-based identification systems can (on the basis of the image scanning evaluation method adopted) be categorized into three distinct types.

1. Contact with pins: This method makes use of ”scanning hand fixation images,” i.e., a set of pins that define the position of individual fingers. The hand is laid on a flat surface, which creates a contrast to the surroundings. The method makes evaluation rather simple, even though it is less comfortable for the user, with some unwanted deformations. In the past, “contact-with-pins” was mainly used in research identification systems. Today, it finds a commercial application. In a study by Reference [35], the authors used five pins for the fixation of the position of the hand before scanning from the top and the sides. For the purpose of the evaluation, 16 geometric characteristics were utilized, which yielded an EER of 6%.
2. Contact without pins: In this case, the hand is freely laid down onto a contrasting surface or scanner. The absence of pins implies a free movement of the hand, which eventually settles in its natural shape. This way, unwanted deformations are eliminated [36]. In their work, [36] the researchers adopted a tabular scanner without pins fixed on it.
3. They evaluated the size and geometry of the finger tips, reaching an FRR value of 11.1% and a low FAR value of 2.2%. These values differ significantly from those obtained when pins are fixed (FRR value of 4% and FAR value of 4.9%). As such, “Contact without pins” is considered the most suitable for security applications due to the importance attached to FAR within domains of the method.
4. Contactless scanning: This method does not require pins or surfaces where the hand will be laid and is, by far, the most user-friendly technique of all. To create contactless scanning points, a standard 2D camera or 3D digitizer is used for scanning. Reference [37] carried out hand scanning in front of a camera in an open surrounding. Evaluation was deliberately fixed at the hand’s center of gravity. As such, it was possible to create homocentric circles that intersect with the fingers. The author measured the fingers using these circles, with the results formed by measuring the size of the fingers in 124 points. FRR and FAR were recorded as 54.3% and 8.6%, respectively, with hand movement and its inclination to the camera surface causing some problems. Oftentimes, the movement from and to the camera leads to significant distortions in the size of the image generated. Instability of the surroundings is another problem associated with contactless scanning, which influences its measurement.

A novel way to identify or verify a person’s identity is by detecting the distribution of veins around the wrist or on the palm surface. The main advantage of this approach is the difficulty in replicating or fabricating the human vein, since the vessels are hidden in the body. References [38] described two possible scanning methods in this regard, which include far infrared (FIR) and near infrared (NIR) [39,40]. FIR is a technology that scans thermal radiation with a wavelength of 15 to 1000 μm of the object (an individual’s wrist or palm surface) under examination. A number of external and internal conditions influence FIR. For instance, temperature and/or moisture of the external environment as well as existing health conditions of the scanned individual can influence its sensitivity. Consequently, the scanned image may be unreliable. NIR on the other uses infrared radiation of the wavelength range of 0.76–1.4 μm of the scanned tissue(s). The technology has a penetration range of approximately 3 mm. Deoxidized hemoglobin found in the veins has the tendency to absorb maximum radiation with a wavelength range between 7.6 × 10^−4^ mm [41]. IR (Infra Red) radiation absorption increases with large veins as compared to tissues adjacent to it [42,43]. As such, the contrast between the object under investigation (large veins) and surrounding objects (tissue) is easily accomplished. Two essential conditions for effective near infrared biometric technology are appropriate camera with IR filter and suitable lighting of the area that uses the IR radiation source. Furthermore, a primary benefit of NIR technology is that the external and internal conditions do not affect the image scanning process. The process is also not affected by skin deviations and tones of the image.

**Figure 1:**
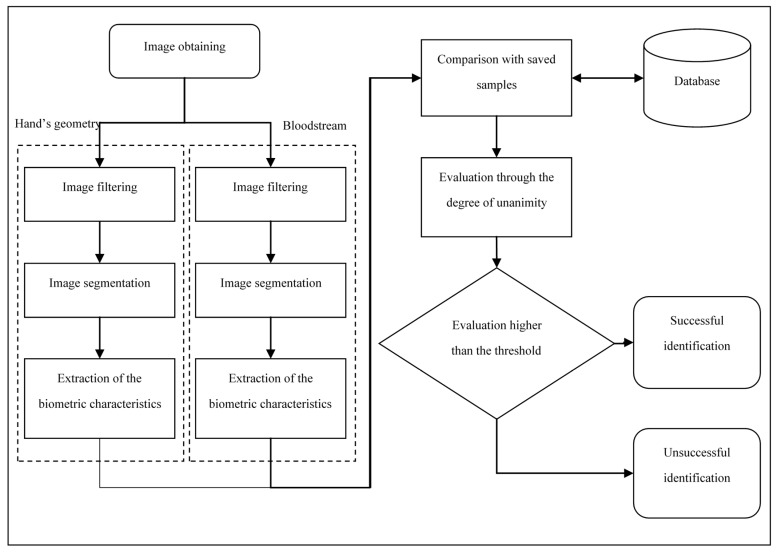
Schematic representation of the proposed identification and verification system.

In the light of the foregone analysis on biometric verification and identification, the current study aims at designing an experimental multi-biometric verification system, which is based on two biometric characteristics: hand geometry and blood flow from the upper part of the hand. Since it has been established in literature that a combination of two biometric features provide better verification and identification results. The proposed system has been selected on the basis that it is impossible to integrate the appraisal on coarse data level (sensor-level fusion) as well as on features absent on a collected biometric characteristics level (Feature-Level Fusion) [6,44]. Additionally, due to the vast misunderstanding between identification, verification, and authentication biometric devices [45], it is important to clarify that the proposed system is a verification system, which checks a user’s biometric identity against those of a number of persons within a database (Figure 1) [45,46,47].

## 3. System Description

The proposed user identification and verification system is comprised of a software that causes it to function according to the steps illustrated in Figure 1.

In this section, the extraction of hand and bloodstream data is discussed. The entire system commences with obtaining the image of the hand, and then that of the bloodstream (vascular structure) [48,49]. Next, background noise is removed from the images to make it fit for further processing. This process is followed by the first screening process (segmentation) for which every aspect of the scanned object is carefully verified by the system, which tries to match the image with existing or similar saved features (images) within the database. Extracted data from the image moves into a central database where the verification is completed. Next, the score-level fusion is obtained for the image (degree of unanimity) and, depending on the threshold, the user is either successfully verified or not. However, this is often done after the data has been normalized as a function of the number of tests carried out. The rest of this section is described in deeper terms and the different blocks are represented in Figure 1. Furthermore, some useful metrics such as EER is often calculated in order to have a full grasp of the efficiency of the system in comparison to other existing systems.

### 3.1. Hand Geometry

#### 3.1.1. Image Pre-Processing

In order to reduce the noise in the image results generated by the system, a basic averaging method (a filtering technique) was used [50]. Although the method does not completely eliminate blurring especially at the edges, it works well for most parts of the image surface. Continuous application of the filter blurs out the entire image. However, once the filtering is executed to a level where it produces a preferred image (an image that is not multi-colored), the filtering process is stopped. The color of the image represents the mean values of the image, which is derived from using a convolution method of calculation. Consequently, the size of the convolution core acts as the variable (it has been identified as the parameter used in the method and the blurring effect is directly proportional to the growing size of the core) and the values are equivalent to 1.

The equation for calculating a 3 × 3 sized core is shown below.

(1) $$f\left(i,j\right)=\frac{1}{9}\sum_{k=-1}^{1}\sum_{l=-1}^{1}g_{}(i+k,j+l)$$

#### 3.1.2. Image Segmentation

The image that is produced by the proposed system is segmented into components with similar characteristics. Segmentation aims at distinguishing scanned objects from one another, as well as from other objects within the surrounding [51]. A variety of methods are available for segmentation. For this study, the thresholding methods has been adopted due to its simplicity in its calculation [52]. Thresholding produces a complete segmentation of objects based on the transformation of the input image *f* on output binary image *f′* according to the relationship shown below.

(2) $$f^{\prime }\left(i,j\right)=\begin{array}{c} 1\;for\;f(i,j)\geq T\\ 0\;for\;f(i,j)<T \end{array}$$

where *T* is in an advance defined constant (threshold) and *f*´(*i,j*) = 1 for the image parts of the examined object. Thresholding also helps in testing the elements of an image in a progressive manner, by assigning values to elements of the image in accordance with identified requirements. Nevertheless, this segmentation method is flawed on the ground that selection of an accurate *T* (threshold value) may be difficult. To automatically set the brightness of the image generated by the system, a “global or local characteristics of the image” can be used. The global image characteristics method uses the information from all pixels in the image in order to determine the threshold value. The threshold is subsequently adjusted in accordance with the histogram generated or the mean value of the intensity of each image point obtained within the image [53]. On the other hand, the local image characteristics emphasize the usage of different thresholds for each element of the image, so that the threshold value is then calculated from the surrounding. The pixel value is calculated by using the following formula.

(3) $$f^{\prime }\left(i,j\right)=\begin{array}{c} 1\;for\;f(i,j)\geq (\mu_{ij}-T_{g})\\ 0\;for\;f(i,j)<(\mu_{ij}-T_{g}) \end{array}$$

where *μ_ij_* denotes an average value of all points from the surrounding. The surrounding size selection is done in accordance with the selected target of the segmentation, and it is mainly dependent on the size and shape of the examined object. *T_g_* is a constant, whose value is dependent on the threshold value, and, therefore, it adjusts accordingly. This constant is often a positive number, but can also become negative depending on the prevailing situation. Segmentations are often selected with respect to the required results. As such, they are empirically identified for a given task.

The software system of the proposed identification and verification was used to experiment both image brightening techniques thresholding [52,54]. Solutions produced using the local image characteristics was not selected due to non-homogeneity of the examined object (hand). This non-homogeneity is caused, for example, by the wrinkles on the skin, different color of the skin, etc. Unfortunately, it was not possible to set up the thresholding parameters in the same way as the size of the surrounding, and the *T_g_* constant. If it were possible, the resultant image would have been subjected to further processing (for determining the contour of the hand). As a result, the global threshold setting method has been adopted, with the average value of complete image intensity reduced by the constant. This method provides the best image that can be subjected to further processing.

#### 3.1.3. Definition of Biometric Characteristics

**Figure 2:**
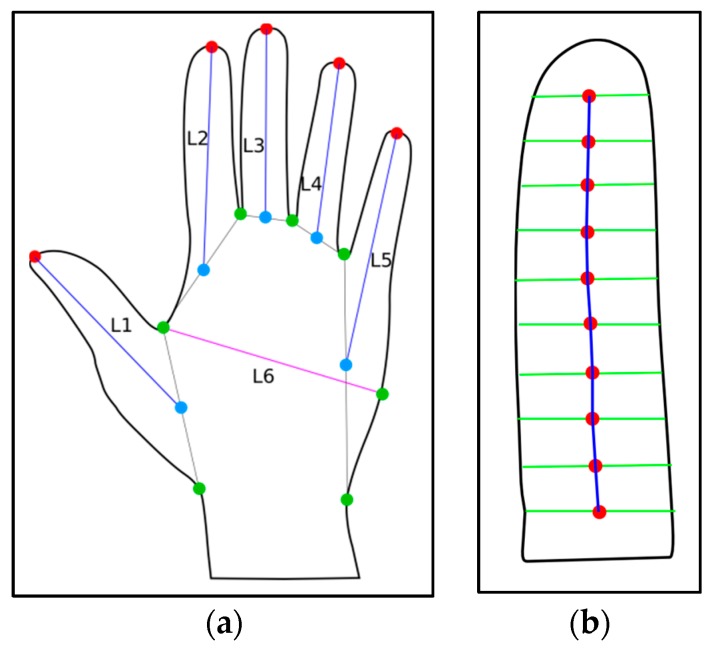
Depiction of measured biometric characteristics [9], where at (**a**) graphics shows the measurable characteristics of the palm of the hand and (**b**) described the base as the point of intersection of green lines.

In order to propose the use of biometric characteristics from this experimental investigation, the studies by References [27,36] were reviewed. The measured characteristics identified for this study, which is utilized in the design of the biometric device, are discussed and illustrated in Figure 2.

Figure 2a shows the measurable characteristics of the palm of the hand. The individual length of the fingers would be measured from the red point (tip of the finger) to the blue point (the central part of the base of the hand). The base is described as the point of intersection of green lines, known as the neighboring values, as illustrated in Figure 2b. Furthermore, the distance between the fingertips are measured (excluding the distance between the thumb and the finger next to it). This is due to the huge variation in the size of the distance between both fingers. Experimenting this on the proposed scanner shows that the difference in the distance from single user images were found to have a greater value than the differences among other users. The distances between points “values” are also measured, but without the thumbs. The width of the palm, L6, is the last measured feature. For a single finger (Figure 2b), there are 10 points distributed along the finger length. The central red point position is determined from the angle located between the sections (represented by blue vectors).

#### 3.1.4. Extraction of Biometric Characteristics

The result of segmentation comes in the form of a binary image (with a background value equal to 0 and the objects value is 255). An algorithm is used for searching the described characteristics of the examined object. Extraction of characteristics is essential to conserve data volume, and obtain higher processing speed. The extraction algorithm, in itself, is responsible for locating the hand contour, which is equivalent to the tested shape of the hand. Finding the contour is quite simple since the wanted line of the related point is visible from the binary image. Working of the biometric characteristics’ extraction algorithm is a function of the contour found. The extraction algorithm causes the localization of the convex contour case, which is responsible for detecting the points on the finger tips. The convex contour case is a polygon, which contains the outermost points of the contour. Hence, the result is formed by a point sequence that creates the case. Consequently, the algorithm searches for the points in the values between the fingers.

**Figure 3:**
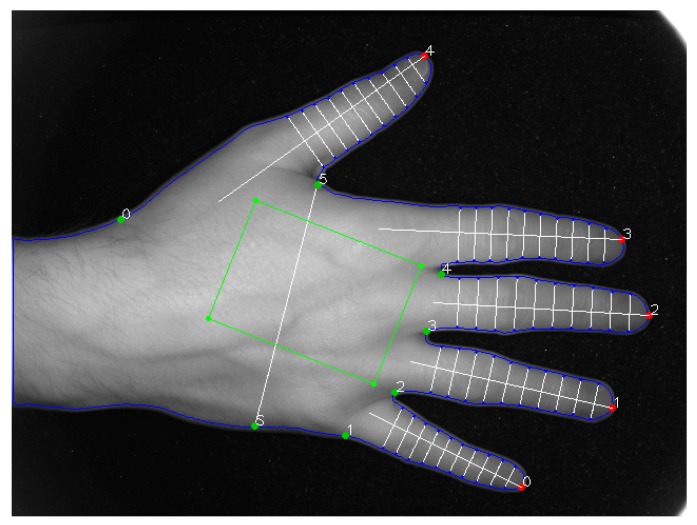
Depiction of all measured characteristics of an object (the human hand).

The contour of the hand is again used for locating defects. Defects represent any point that is located on the contour, which is present on the part demarcated by the surrounding points. At least one defect is prevalent in between any pair of points. This follows the pattern of a convex case. Hence, the identified defects are filtered to a minimal depth (distance from the case), which eliminates a number of small defects. The only defects left are often the ones corresponding to the values between the fingers. In the next step, measurement of the biometric characteristics takes place, as displayed in Figure 3. The characteristics for each finger, and the overall characteristics of the whole hand are measured.

### 3.2. Bloodstream

#### 3.2.1. Image Pre-Processing

**Figure 4:**
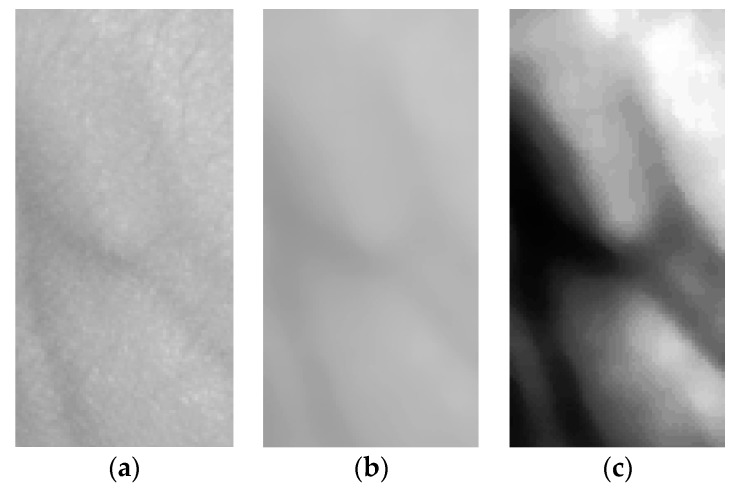
Process of bloodstream filtration: (**a**) before filtration (left), (**b**) after median filtration (middle), and (**c**) after equalization (right).

In the identification and verification of blood flow at the upper part of the hand, the first stage is to locate the point of interest (ROI). It is identified as the part of image that has the object of interest. In this context, the object of interest is the bloodstream at the upper part of the hand (this is based on a pre-selected biometric characteristic). To define the location and the value of ROI, the position and the shape of the hand needs to be identified. Extracting blood stream biometric characteristics implies gathering information on the biometric characteristics of the hand. Figure 4 shows how ROI is derived (green rectangle). The ROI area starts from the joints level, and is associated with the vector that exists from finger valley point numbers 2 and 4, and up to the middle of the hand. This helps to differentiate (through ROI width) each user and/or specific individual image. The height of ROI is determined and expressed as a multiple value (precisely in multiples of 1.4) of the width.

During the second phase, the ROI is used to develop a copy of the new image, which is subjected to rotation. The goal of image rotation is to ensure that the orientation of the bloodstream image is vertical and corresponds with the longer side. Figure 4 shows the result after copying ROI (Region of Interest) and rotating the image.

Rotation and equalization are followed by image filtering. In the context of the proposed system, the image is filtered using a median filter with a relatively large surrounding of the points (11 pixels). As such, significant image smoothening is achieved, so that skin wrinkles and body hair distortions are removed. However, the shape of the veins is retained on the image (Figure 4b).

To further treat the images, equalization of the histogram was carried out. This process adjusts the brightness of individual pixels relative to the histogram. For the images with a similar intensity of pixels, as in this case, the contrast becomes better. Using this procedure, the difference between the background and the object of interest (bloodstream) increases (Figure 4c).

#### 3.2.2. Image Segmentation

**Figure 5:**
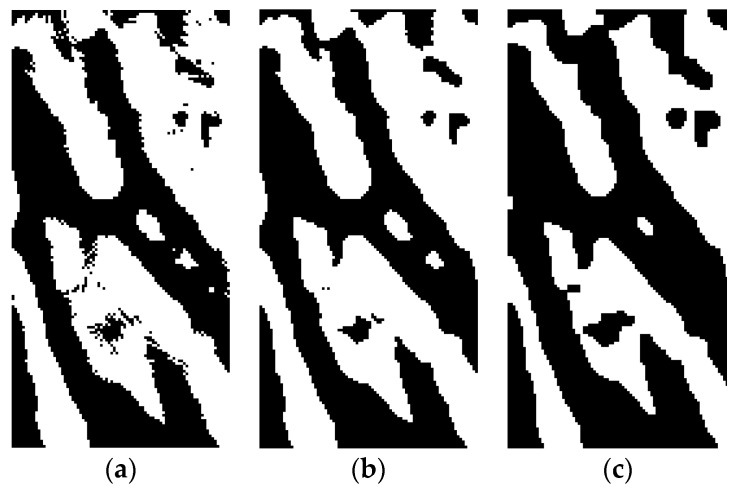
Process of bloodstream filtration: (**a**) after thresholding (left), (**b**) after median filtration (middle), and (**c**) after dilatation and erosion (right).

The adaptive thresholding method was used for segmentation of the bloodstream [13]. The obtained image after thresholding (Figure 5a) corresponded to the bloodstream of large veins. Unfortunately, the image bears plenty of noise, with several unfinished lines, and calls for filtering.

The median filter in combination with a morphologic filter was used. The median filter of 3 pixels mainly removes the small fragments in the image (Figure 5b). Furthermore, dilatation and erosion processes are carried out, so that some sections of the individual parts of veins connect. These were previously not connected due to noise and faults in the image (Figure 5c). The next step in the image processing is the extraction and evaluation of biometric characteristics of the bloodstream results. This is derived by comparing the obtained image and the image of the fingerprint during dactyloscopy, as discussed by Reference [20].

**Figure 6:**
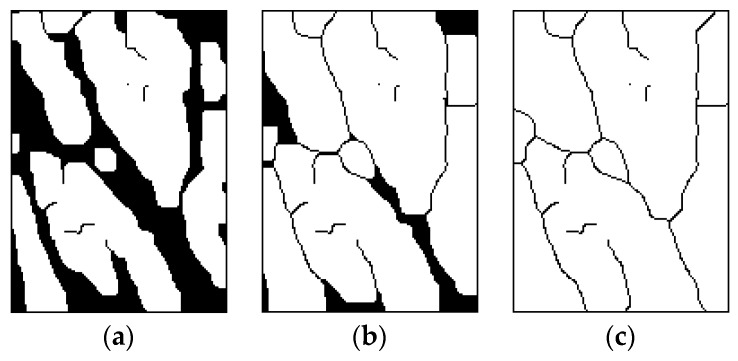
Process of thinning: (**a**) 2nd. Iteration (left). (**b**) 6th. Iteration (middle). (**c**) Result (right).

After the smoothening and thinning of the image, the skeletonization of the image is automatically achieved (the skeleton is extracted) [2]. Thinning is a morphologic operation that is responsible for the deletion of selected pixels from the binary images. The process shares similarity to opening and erosion. During thinning, pixels at the edges get deducted from the objects, but not in such a way that disturb the object results. Iterative thinning can be achieved using an algorithm. In 1986, Alberto Martin and colleagues [55] analyzed a few different thinning algorithms. The results of their study indicated that the best outcomes from the point of reliability and effectiveness are achieved by algorithms that are based on the method of samples and the method “sign and delete.” One of the representative algorithms of this group that was chosen for this study is the thinning algorithm by Zhang-Suen [56].This thinning method is simple, and gives room for evaluation even when in low-quality contour objects. Figure 6 shows the process of thinning iterations.

As noticeable on the last image (Figure 6c), due to the faults in the image, parts of the skeleton as well as the points or unfinished lines are still visible. These are veins that, in one place, run deeper into the tissue of the hand. Therefore, they become invisible for the camera. It is often necessary to eliminate such artifacts before the extraction.

**Figure 7:**
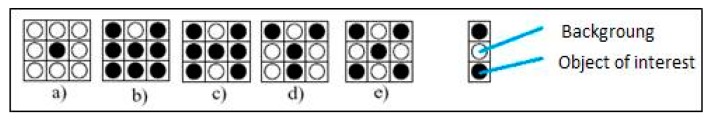
Examples of the number of pixel connections.

**Figure 8:**
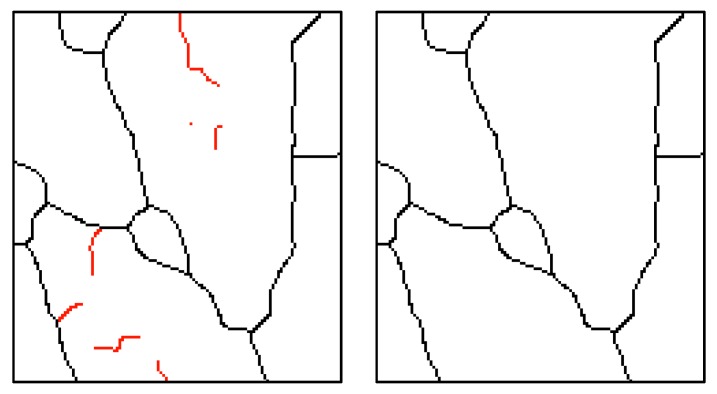
Skeleton before and after filtration.

For the elimination vein line artefacts, a self-created image filter was used. This filter is based on the number of the pixel connections. This is the combination of the number of individual connections of the examined pixels and the chosen object’s pixels. The number of such connections may have values between 0 and 4. Figure 7 shows examples of the surrounding pixels. The filter’s algorithm runs through all image pixels and searches for pixels that are at the end of the line (stand-alone pixels). The number of connections is usually 0 or 1 (Figure 8).

#### 3.2.3. Definition of Biometric Characteristics

The definition of the biometric characteristics for the verification of bloodstream results originate from the work [20]. For the definition of biometric characteristics in the context of this paper, the similarities of the bloodstream and fingerprints were used. In order to verify a person according to the fingerprints, the comparison of the critical point position (*minutiae*) is used instead of carrying out a comparison of the whole image (on the basis of the sample). For fingerprints’ evaluation, the minutiae is used at the beginning and at the end of a dermal papillae, bifurcation (decoupling), hook, and eye. Within this experiment, two types of minutiae were defined, which includes vein branching and vein ending at the top portion of the image. Quantity of located minutiae is expected to be different across all test subjects. However, for one user, the quantity of minutiae located during each scan should be the same. Moreover, the positions of these minutiae in the case of one user should be expected to maintain the same position, no matter the number of scanning. This is because all subsequent images are compared to the image on which the extraction of bloodstream biometric characteristics was done. Therefore, the experimental software works with the minutiae’s coordinates, which are closely related to the image of the bloodstream.

#### 3.2.4. Extraction of Biometric Characteristics

In order to extract minutiae from the images, we adopt the principle of the number of pixel connections. The algorithm scans the bloodstream skeletal image to go through each of the pixels, which is the property of the object of interest (ROI). The number of pixel connections, which is measured as the pixel of the object of interest, is scanned. Pixels with higher connections values (greater than 3) are responsible for creating a branching point, which is, otherwise, referred to as minutia. Pixels whose connections are equal to 1 and are found at the edge of the image are also marked as minutia and become the end point of the vein.

### 3.3. Evaluation Using a Degree of Unanimity

#### 3.3.1. Calculation of Fractional Metrics Evaluation

**Figure 9:**
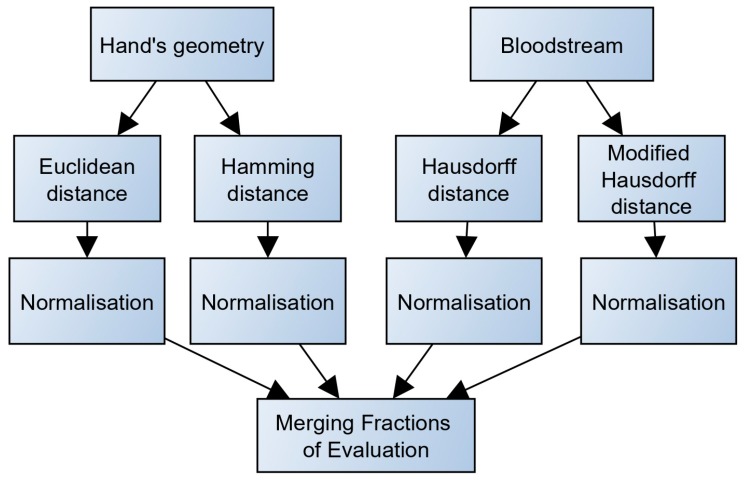
Calculation of fractional metrics evaluation.

In the proposed experimental project, a number of methods were used for calculating score-level fusion [57] (Figure 9).

▪ Euclidean distance, which is the distance measured between two points, is located in the ‘*N*’ dimension. The Euclidean distance is computed using the formula below.

(4) $$d=\sqrt{{\sum_{i=1}^{N}\left(x_{i}-t_{i}\right)}^{2}}$$

where *N* is the number of dimensions, which is (in the case of a template and testing data) the number of measured biometric characteristics, *x_i_* is the *i*-th element of the tested data, and *t_i_* is the *i*-th element of the template.

The number of template dimensions is equal to one testing data. The resulting value is the addition of all differences between the template and testing data.

▪ Hamming distance: This is another way of computing the score-level fusion. Hamming distance originates from the theory of information. In comparing two chains with the same length, the Hamming distance shows the lowest number of differentiation positions. In other words, it presents the number of substitutions that need to be established in order to change one chain into the second one. In their study, the researchers [6] generalized Hamming distance into a form suitable for evaluation of biometric data similarities. The authors suggested the use of a comparison on the basis of the number of non-unanimous biometric characteristics. The result is formed by a metric that does not measure deviation as in the case of Euclidean distance. Rather, it indicates the number of individual biometric characteristics for which there are higher deviations (during the comparison of the testing data and the template) than the root mean square (RMS) error of biometric characteristics. The RMS error is defined for each feature during template generation. Such an error is selected due to the presumption that the characteristics of one user during multiple photographing would never be completely identical. The presumed allocation of values for the given characteristic corresponds to the normal allocation. Hamming distance is calculated according to the following formula.

(5) $$d(x_{i},{\bar{x}}_{i})=\#\{i\in \{1..N\}/|x_{i}-{\bar{x}}_{i}|>\sigma_{i}\}$$

where *x_i_* is a biometric characteristic of the testing data with a serial number *i*, ${\bar{x}}_{i}$ is the average of the biometric characteristic (from the template) with the serial number *i*, *N* is the overall number of biometric characteristics for the given template, and $\sigma_{i}$ is the RMS error (from the template) with the serial number *i*.

▪ Hausdorff distance [58] is another method useful for a score-level fusion calculation. It determines the distance between two set of points found in the metric space. In simple terms, the two data sets that are in closest proximity to one another such that points of the second dataset can be found near the surroundings of the first dataset. Hausdorff distance - Helicity Violating (HV) is considered to be the longest distance among the existing distances between the set of points. It is created by joining one point of the first point set to another point on the second set and vice versa. If there are similarities between two sets of points, then HV has a lower value. Since the biometric characteristic of the bloodstream is composed of various sets of points, such a position on the image is essential. In this experimental set-up, HV is able to calculate score-level fusion, which naturally compares the similarity in shapes. HV is, however, flawed on the sensitivity to remote values. Oriented HV, which is marked $\bar{H}$ between the sets of points *A* and *B*, corresponds to the maximum distance from all pairs x∈A and y∈B. The oriented HV is expressed by the equation below.

(6) $$\bar{H}(A,B)=\max_{x\in A}\{\min_{y\in B}\{\|x,y\|\}\}$$

where ‖, ‖ is a random evaluation function, which is mainly a Euclidean distance.

The oriented HV is asymmetric. Therefore, $\bar{H}(A,B)\neq \bar{H}(B,A)$ applies. It also does not provide the distance between the sets *A* and *B*, but only provides the longest distance from the point x∈A to the closest point y∈B. On the other hand, the non-oriented HV, which is marked *H*, is the maximum from $\bar{H}$ in both directions, and indicates the difference of the two sets of points. The formula for the calculating non-oriented HV is shown below.

(7) $$H(A,B)=\max\{\bar{H}(A,B),\bar{H}(B,A)\}$$

▪ Modified Hausdorff distance: As earlier mentioned, HV is very sensitive to distant values. This implies that even a few points from the testing set of points that are outside of the template points would cause a large increase in the value of HV. This is regardless of whether or not the sets are very similar to each other. In order to find the solution to this weakness, researchers [58] looked at many different modifications of HV. Results from their analysis showed that, while using modified HV (further MHV), the problem of distant values is suppressed. In contrast to the previous formula, the non-oriented MHV can then be defined as:

(8) $$H(A,B)=\frac{1}{N_{A}}\sum_{x\in A}^{}\min_{y\in B}\{\|x,y\|\}$$

where *N_A_* is the number of elements in the set *A* and ‖. ‖ is a random evaluation function, using mostly Euclidean distance.

#### 3.3.2. Normalization of a Fractional Metric

Before merging the results of the individual metrics, it is necessary that the results undergo some form of normalization. Individual metrics provide results in different “dimensions.” Normalization within this study is carried out using a ‘min-max’ method within the experimental software. It is calculated according to the formula below.

(9) $$no=\frac{o-\min_{i=1}^{N}o_{t}^{i}}{\max_{i=1}^{N}o_{t}^{i}-\min_{i=1}^{N}o_{t}^{i}}$$

where *o* is a coarse evaluation, *N* is the number of elements in the set of testing data, and $o_{t}^{i}$ is an element of the testing data.

#### 3.3.3. Merging Fractions of Evaluation

The merging procedure in multiple biometric systems (blending of scans and results of different types of biometric characteristics) is carried out in different levels of processing. In the multi-biometric scanner proposed within this study, merging is done by recording fractional results from individual metrics. In this method, individual conformity assessments are combined after normalization. This method is most commonly used [59,60,61] while it provides clear and simple results processing.

In order to calculate the overall evaluation, it is first necessary to normalize the individual outputs from different metrics. Normalization ensures that all intermediate results have the same weight regardless of the method used.

The metrics merging itself is done using an arithmetic average. Furthermore, implementation is made possible. At the same time, the process provides the best results [62]. During the final verification phase, the template is tested with the best score-level feature. If the score level fulfills the requirement of the threshold (set to 50% in this case), then the process is tagged successful. If not, then it is tagged unsuccessful.

### 3.4. Image Scanning

#### 3.4.1. Proposal of the Scanning Device

**Figure 10:**
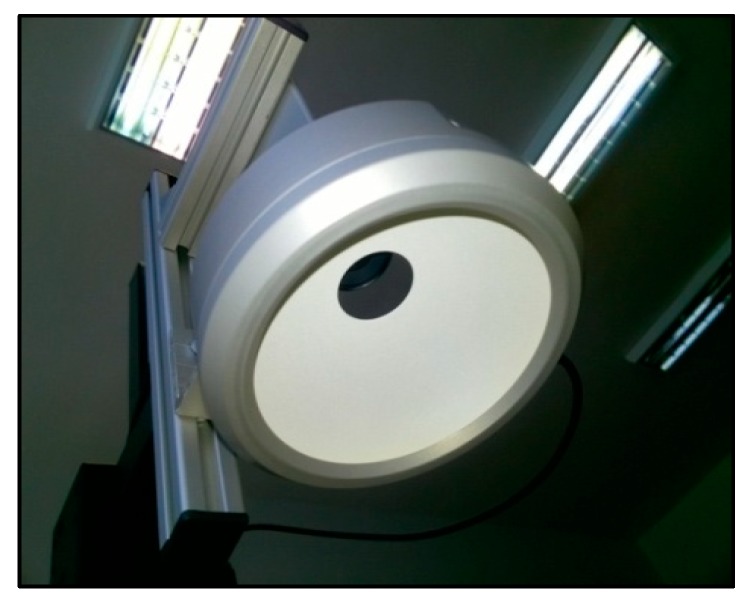
Configuration of the camera and lighting.

**Figure 11:**
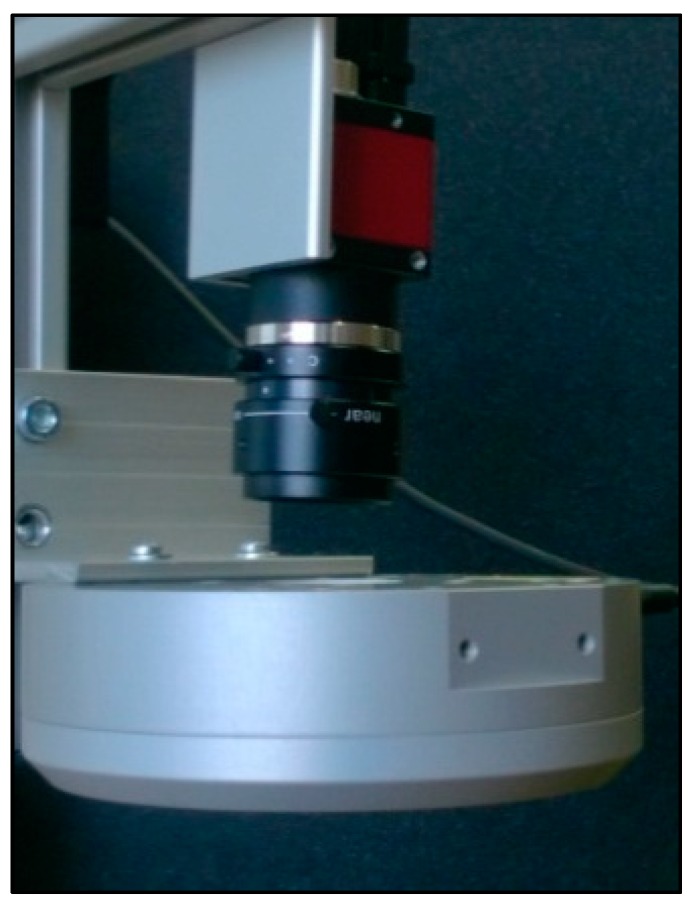
Configuration of the camera and lighting.

**Figure 12:**
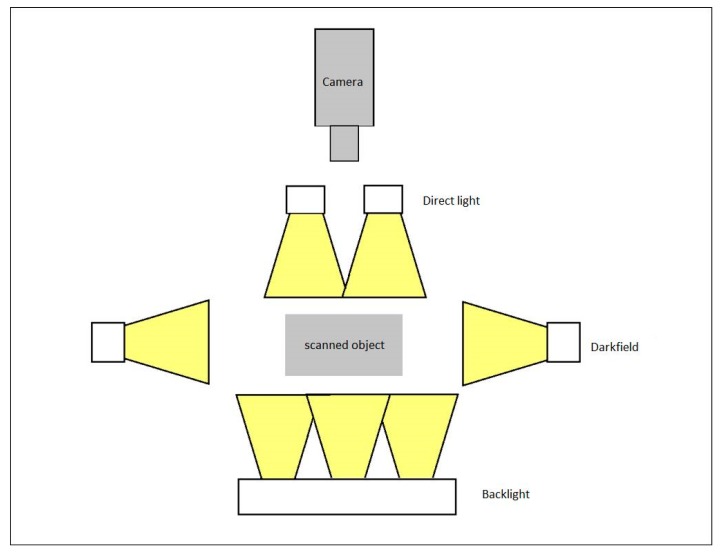
Possible types of lighting within the scene.

Image scanning is the first step toward the experimental implementation of the multi-biometric scanner. Effective image scanning positively influences the results to a great extent, especially during the image evaluation. To arrive at an appropriate image that will be subjected to further processing, configurations such as background lighting, direct lighting, and side lighting can be used (Figure 10, Figure 11 and Figure 12). The image selection process is based on the task requirements. For instance, background lighting is considered to be an ideal option to measure the shape of the object. This is because it highlights the contour of the object (hand). In the context of the current work, direct lighting configuration was adopted.

For effective image processing, suppressing background noise (influence of the surrounding) is a very important requirement. Background noises negatively affect image processing and further assessment. Nevertheless, the use of additional lighting can resolve this problem. Additional lighting creates an improved scene and looks like an industrial light. The use of a filter that allows the passage of radiation only has the wavelength equivalent to that of the light in use, which also improves the process. A lighting requirement includes:

- Sufficient intensity
- Homogenous lighting of the scene
- Consistency of the light intensity over time (to guide against depletion).

Due to these lighting requirements, experimental design and implementation of the multi-biometric scanner in this paper adopted an industrial type of lighting for the hardware component of the scanning device. This was needed to achieve the required homogeneity associated with further image processing. For the same reason, the proposed scanning device has been equipped with a special camera. This camera will, however, not function based on automatic corrections of the image as compared to commonly available cameras. Table 2 summarizes the approximated prices of some components of the proposed scanner.

#### 3.4.2. Components of the Scanning Device

A digital camera with resolution of 640 × 480 pixels and 8 bits was used for image scanning. This suggests the realization of grey-toned images, with 255 shades of grey. Firewire interface was used to transfer stored data from the camera. A stable focal distance of 4.5 mm from the manufacturing class VCN was used. The IR filter plays the important role of scanning the IR part of the spectrum [63,64,65]. This was needed since sunlight impact on the scanned image is a limitation. Another part of the scanning device is the source of IR radiation. Therefore, tests with different sources of IR radiation were conducted. The lighting tests include: direct circular lighting, direct linion lighting, diffused DOM lighting, and background lighting. On the basis of the test results, the most suitable lighting, DOM, was selected. This lighting uses LED lights with the wavelength of 850 nm and allows for generation of images with homogenous lighting, with a well recognizable structure of veins on the top of the hand, as well as a sufficient contrast of the hand against the background. This is good for further evaluation of the hand contour.

Lights and the camera configuration was put on the construction from aluminum profiles, which was mounted on an adjustable tripod (based on the configuration). The background comprises of a black matte surface that helps to get the contrast between the background and the scanned image of the user hand. The whole configuration of the camera, optics, and the lighting is displayed on the images in Figure 10 and Figure 11. From these estimates, the average cost of the scanner is about €1490, which is relatively expensive. Nonetheless, selected sellers/manufacturers are the most revered in terms of sales of most durable spares, which is why the prices are so high. The components may be purchased from other sellers at cheaper rates, which will most likely reduce the average possible cost of the proposed scanner [66].

## 4. Results: Testing of Developed Application

### 4.1. Testing Methodology

For the purpose of testing the biometric verification system proposed in this paper, 280 (40 subjects, where each provided seven images) images of the hands of different persons was used. Although this value is small compared to existing works by References [27,29,30,31,32,33,34,67], this was mainly due to the unavailability of the scanning system as the ones used were gotten on a friendly loan. Participants consist of males and females between the ages of 25 to 60 years old. Characteristics of the tested persons is summarized in Table 3 and Table 4. Two unique experimental design pathways were followed to check the behavior and evaluate how the proposed method progresses. First, the hand geometry was verified by Hamming distance, and then the bloodstream using MHV.

To establish a scientific experimental backing for the current study, it was vital to check the proposed experimental set-up to those in some existing works. As seen in the experimental design from Reference [28], based on the number of test subjects within this experiment, observations show that data associated with the bloodstream are not very sensitive to temperature when the human body is at rest. While Reference [28] noted that perfusion of blood was better in their study, ours yielded a different result since hand geometry seems to be better in terms of the rate of recognition. In Reference [34], the authors explained in their paper that matching scores are derived from the process of triangulation and binarization using vein structures, and from the distance of knuckle points. The current experimental procedure also follows the route of binarization, but with a convex contour polygon for point location detection. A huge part of this experimental procedure follows the ideas of Reference [33]. The pair worked on finger vein and geometry of the image characteristics. Just like in the case of our experiment, segmentation was done through the location of lines at different finger valleys and linking it to the center of the palm through a convex polygon [33]. This was closely followed by locating feature points through which extraction takes place. By adopting the calculation of the Hamming distance, we distinguish between the feature points of an enrolled and input image respectively.

Forty persons were selected for the testing due to the limited number of available hardware components for image scanning. Each had a hand image tested seven times. The scanners were borrowed only for a limited period of time. As such, only a few testing data was generated. The testing of the system was done on a personal computer with the following parameters (only the parameters that may influence the running of the multi-biometric software are mentioned).

- OS—Windows 10, 64 bit
- CPU—Intel Core i5-527U with frequency of 2.7 GHz (maximum turbo frequency of 3.1 GHz)
- RAM—8 GB

### 4.2. Tests of the Experimental System Speed

Time taken to evaluate a tested image is between 0.6 and 0.9 seconds. In terms of calculations, it was somewhat difficult to derive the algorithm for the extraction of biometric characteristics. Table 4 summarizes the processing speed of the individual program steps and the corresponding image evaluation.

### 4.3. Results

**Figure 13:**
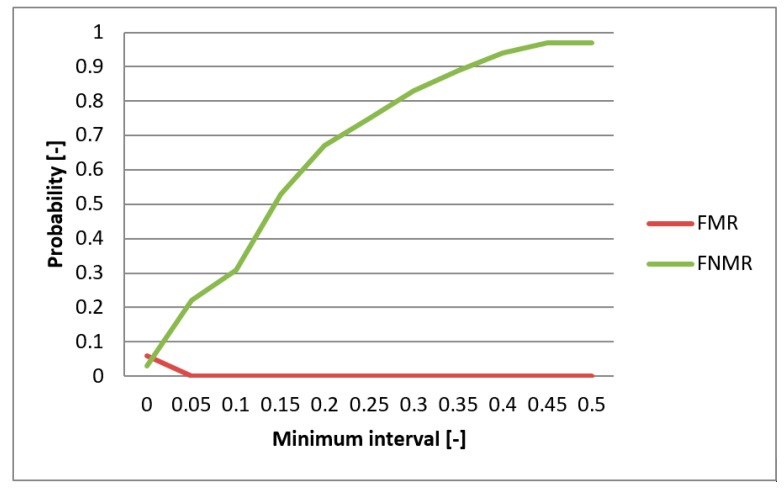
EER for united metrics of Hamming and MHV.

**Figure 14:**
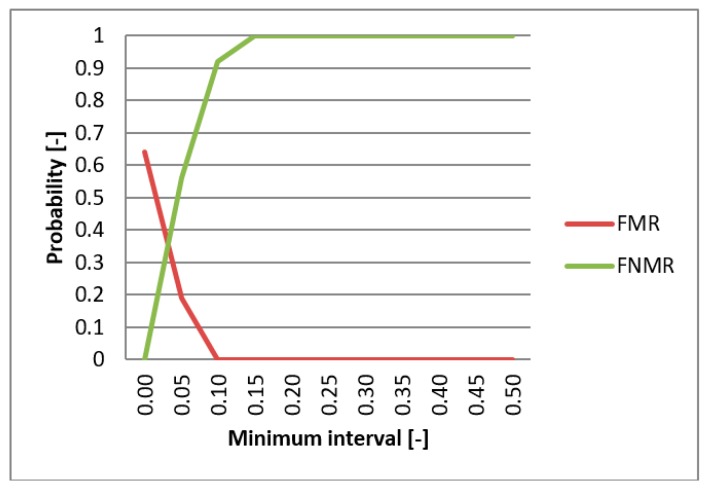
EER for Euclidean distance.

**Figure 15:**
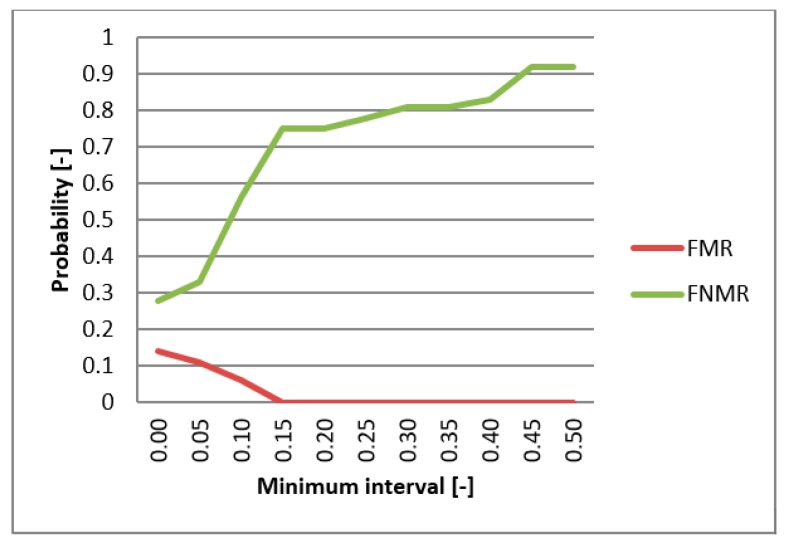
EER for Hamming distance.

**Figure 16:**
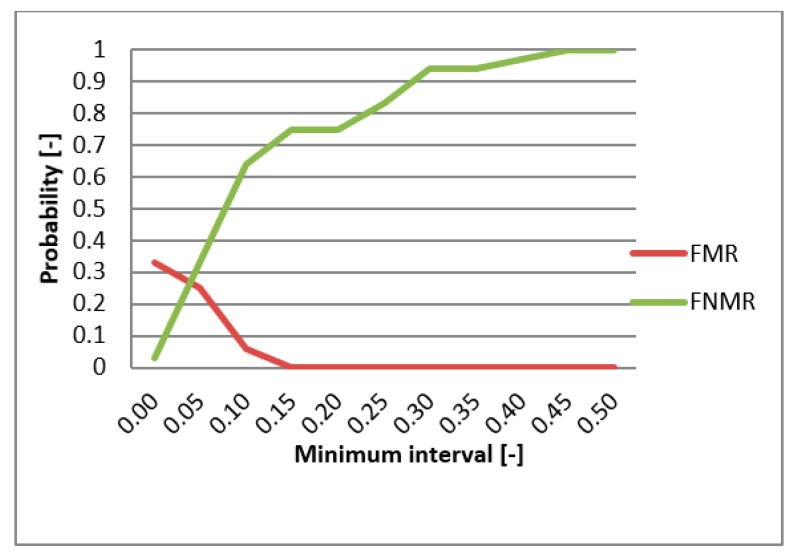
EER for HV.

**Figure 17:**
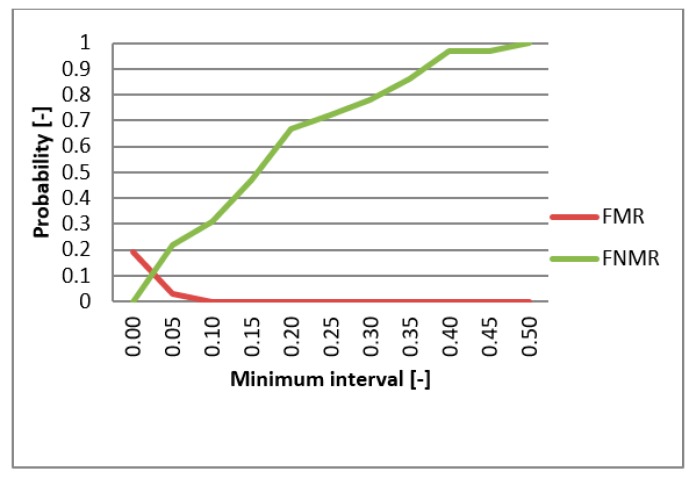
EER for MHV.

FMR (False Match Rate), FNMR (False Non-Match Rate), and EER functions were utilized in evaluating the biometric system. These functions specify the faults frequency of the system. First, the comparison of the individual metrics for the data gathered from participating persons were compared for single-biometric and multi-biometric systems. The threshold value was chosen to be 0.5 [-] and the boundary of the minimum interval varied from 0 to 0.5 [-] (Figure 13). For the metrics normalization, the previously min-max method was applied. The graphs presented in Figure 14 and Figure 15 capture the test results of the two different metrics for hand geometry. Graphs in Figure 16 and Figure 17 capture the test results of the two different metrics for the bloodstream.

In the next part of the testing, the best metric for hand geometry (Hamming metric) as well as the best metric for the bloodstream (MHV) are selected. With these metrics, the multi-biometric system was created and the performance was tested against the data described above (Table 5). The results are shown on the graph in Figure 13. As expected, results showed that the multi-biometric system performed better in comparison to a single biometric system. The EER value for the multi-biometric system was found to be half of what is obtained from a single bloodstream biometry. Moreover, the progress of the FNMR fault was smoothened and partially reduced.

## 5. Discussion

The database developed for this study was built using 280 images (finger view and palm view, vascular images and back of the hand) from 40 individuals, i.e., seven images per subject. Two of the images generated per person (Total of 80 images) was used to train available researchers, so that they get familiar with the systems. Nevertheless, these images were carefully derived so that they were also useful for analysis. In testing our verification technique, each of the generated biometric data is technically subdivided (partitioned) into 2 × 50, 1 × 80, and 1 × 100 samples. Individual scores (from each uni-modal) were then summed up to arrive at the overall score. The equal error rate of the blood stream (11%) and hand geometry consolidated with the blood stream (5%). This approach is dependent on score-level fusion with the individual. Score results were normalized to achieve the stated percentages.

In addition to the samples used for training, extracted characteristics per scanned hand is related to how the proposed multimodal system works. The more the number of scanned hands, the more the extracted features and the better the behavior of the proposed multi-modal systems in terms of its performance. The implication of this is that the system witnesses some form of increased computational cost, which is similar to the finding of Reference [68]. As such, the number of extracted characteristics per scanned hand is checked against the EER. As noted by Reference [68], there is a possibility for an increment in the cost of computation in terms of the overall number of extracted characteristics. For example, this cost covers the time needed to input the first set of features into the database, which, thereby, trains the system, in addition to taking it for verification. Verification time is dependent on the speed of the computer (specification) used, as well as the algorithm for the experiment. There are a number of systems with rapid verification, segmentation, and feature extraction times. Some of these features utilize multi-dimensional vectors with more than two biometric approaches. In general, a high number of extracted characteristics imply longer processing times.

A number of challenges were noticed in the course of the experimentation with the proposed multi-biometric device. However, many of these challenges can be resolved at the point of use except for the issue posed by the extraction algorithm, which causes a lengthy time of verification. Algorithms of the individual metrics (hand geometry and blood stream analysis) demonstrates specific speed and precision issues that were noticed during experimentation, and may have significant effects on results. For instance, the low contrast produced by the bloodstream image during the investigation proved to be a limiting factor. There are also problems of discontinuation of veins and shadow produced by the human skin. This shadow image is confused to be another vein. Such small contrasts are most likely caused by a large distance between the lighting of the scene and the scanned hand. Camera resolution of 640 × 480 pixels is another factor that seems to affect results negatively. This produced very small differences in measured widths of the fingers among persons. This problem can, however, be resolved by using cameras of higher resolution to produce more precise evaluation and differentiation results. However, this was not verified in this study due to time constraint. Differentiation and evaluation of individual metrics is done via frequency comparison of the double type faults (false identification) with the EER values. The lower the EER value, the higher the level of precision of the given metrics. The least obtained value was measured for modified Hausdorff distance metric (MHV), which yielded an EER value that was 11%. Furthermore, the best metrics for the given biometric characteristics (Hamming distance and MHV) were chosen and consolidated into the final evaluation, which results in a multi-biometric model with an EER value of 5%. This implies that, as expected, metrics consolidation meant improved system precision.

The biggest problem of the proposed multi-biometric system could be the unwanted influences from the surrounding. For instance, direct sunlight would influence the intensity of the scanned image. A change in features of the platform on which the scanned hand is laid can also influence the results. Lastly, the user-friendliness of the hardware for hand scanning would need to be improved in order to make the usage of the system faster and more comfortable.

## 6. Conclusions, Limitations, and Future Work

This paper proposes the use of a multi-biometric system, which is useful for identification and verification of a person through his/her hand geometry and bloodstream behavior (at the upper part of the hand). The study experimentally tested the effectiveness of the system using a number of research participants. The system proved to be able to verify users with high precision by demonstrating good differentiating abilities. The ERR value of the system is estimated as 5%, which is higher than most existing systems (Table 1). This can, however, be optimized. The proposed system is flawed on the lengthy time taken to carry out the verification. As such, attaining commercial usage would mean optimizing an extraction algorithm for the biometric characteristics. This aspect paves way for future research, with goals of speeding up user identification and verification time. 

## Figures and Tables

**Table 1:** Some multi-modal biometric studies from 2009 until today.

Study Reference	Year	Combined Biometric Methods	Sample Size	Equal Error Rate (%)
Current work	2019	HG and FV	40	5
[27]	2013	FV and HG	100	0.06
[29]	2019	PP and FG	237	58
[30]	2014	HG and FV	204	0.02
[31]	2015	MFV	106	0.08
[32]	2017	FK and FV	100	0.35
[33]	2010	FV and FG	102	0.075
[34]	2009	FV and KS	100	1.14

**Table 2:** Estimated prices of the proposed scanner components.

Component	Seller/Manufacturer	Amount (in €)
CCD camera GuppyPRO F-031B	Allied Vision Technologies GmbH	600
Objective VCN 1.4/4.5 f = 4.5 mm	Vision & Control GmbH	150
IR filter	Heliopan Lichtfilter-Technik Summer GmbH & Co KG	40
Lighting SFD 42/12 IR	Vision & Control GmbH	700
Total		1490

**Table 3:** Division of tested persons based on gender.

Men	Women
80%	20%

**Table 4:** Division of tested persons based on age.

20–29	30–39	40–49	50–60
20%	50%	20%	10%

**Table 5:** Times needed for the execution of individual processing steps and the image evaluation.

Program Step	Hand Geometry		Blood Stream	
	Average Time [ms]	Maximum Deviation [ms]	Average Time [ms]	Maximum Deviation [ms]
Image pre-adaptation	1.25	14.75	3.5	8.5
Segmentation	1.08	8.9	152.7	70.3
Extraction of characteristics	667.5	168.5	8.83	3.26
Calculation of the degree of differentiation and allocation	0.97	3	1	1.9
